# Miniaturized Dual Band Multislotted Patch Antenna on Polytetrafluoroethylene Glass Microfiber Reinforced for C/X Band Applications

**DOI:** 10.1155/2014/673846

**Published:** 2014-06-01

**Authors:** M. T. Islam, M. Samsuzzaman

**Affiliations:** Department of Electrical, Electronic and Systems Engineering, Faculty of Engineering and Built Environment, Universiti Kebangsaan Malaysia, 43600 Bangi, Selangor, Malaysia

## Abstract

This paper introduces a new configuration of compact, triangular- and diamond-slotted, microstrip-fed, low-profile antenna for C/X band applications on polytetrafluoroethylene glass microfiber reinforced material substrate. The antenna is composed of a rectangular-shaped patch containing eight triangles and two diamond-shaped slots and an elliptical-slotted ground plane. The rectangular-shaped patch is obtained by cutting two diamond slots in the middle of the rectangular patch, six triangular slots on the left and right side of the patch, and two triangular slots on the up and down side of the patch. The slotted radiating patch, the elliptical-slotted ground plane, and the microstrip feed enable the matching bandwidth to be widened. A prototype of the optimized antenna was fabricated on polytetrafluoroethylene glass microfiber reinforced material substrate using LPKF prototyping machine and investigated to validate the proposed design. The simulated results are compared with the measured data, and good agreement is achieved. The proposed antenna offers fractional bandwidths of 13.69% (7.78–8.91 GHz) and 10.35% (9.16–10.19 GHz) where S_11_ < −10 dB at center frequencies of 8.25 GHz and 9.95 GHz, respectively, and relatively stable gain, good radiation efficiency, and omnidirectional radiation patterns in the matching band.

## 1. Introduction

Present telecommunication systems aim to use the same device for multifrequency operation and, therefore, require antennas with broadband and/or multifrequency capabilities. In this regard, a microstrip antenna is preferable due to its low profile, light weight, low cost, and ease of integration with microwave circuits [1]. Most of the researchers have focused their efforts on modifying the patch antenna to fulfill these new requirements (multiband and/or broadband). One of the major problems pertains to multimedia and broadening the bandwidth because these operations primarily depend on exciting two or more resonances, which are closely spaced in the case of broadband and far apart in the case of dual or multifrequency operation.

In order to design smaller and compact wireless devices, it is obligatory to miniaturize size in different ways. There are a number of requirements such as wide bandwidth, less expensive, miniaturized size, steady radiation patterns, and consistent gain for multiband antennas (S band, WLAN, WiMAX, C band, and X band). Various techniques, such as etching slots in the patch [2–5], reactive loading and stacked dielectric multilayer [6–10], loading by dielectric resonator [11, 12], ground plane modification [13, 14], fractal shape [15–17], electromagnetic band gap (M-EBG) structure [18], using parasitic elements [19], frequency selective surface [20], and optimization technique [21, 22], can be used to produce broadband and/or multiband antennas. Among the studied wideband or dual band antennas some of them are working for C and X band applications. A slot is designed to act as a parasitic element for higher frequencies to increase the bandwidth and efficiency in [2]. By coupling with the monopole structure, the slot acts as a parasitic element. Nonetheless, the gain theta (*θ*) at the radiation pattern seems to increase more than gain phi (*φ*) when *φ* = 90° at higher frequencies. For X band applications, an S-shaped patch antenna was designed where the authors introduced two slots to perturb the surface current path [4]. The antenna dimension was 30.08 mm × 45.9 mm and the antenna was working at 10 GHz. A dual frequency antenna with two stacked parasitic elements has been designed in [6]. Although the parasitic elements assemble a bulky antenna, the antenna dual frequency bandwidth and gain are increased by controlling the coupling distance with the parasitic elements. Furthermore, low-temperature cofired ceramic (LTCC) technology has been used to design a dual band microstrip antenna for global positioning satellite (GPS) operations [7]. Dual band behavior has been obtained by inserting a small X band microstrip patch antenna into a large L-band antenna [8]. The antenna dimension was 30.08 mm × 45.9 mm and the antenna was working at 10 GHz. Microstrip-fed dielectric resonator antennas for X band applications have been proposed in [11]. A slot antenna and a dielectric resonator antenna (DRA) were combined to effectively design a dual band dielectric resonant antenna for C and X band applications [12], but this was achieved through a complex multilayered structure. A slotted triangular shaped C and X band patch antenna for satellite applications has been proposed with high dielectric and costly material [13]. A ceramic polytetrafluoroethylene composite material-based miniaturized split-ring C and X band patch antenna was designed [14]. The proposed antenna obtained operating bandwidths (reflection coefficient < −10 dB) ranging from 5.0 to 6.5 GHz, 9.1 to 9.6 GHz, and 10.7 to 11 GHz. However, the antenna was designed on a high dielectric and costly substrate. A spirograph planar antenna for C/X band application has been proposed where the number of points increasing in the spirograph miniaturizes the radiating element and increases the impedance bandwidth but the gain is low [16]. A C band antenna using electromagnetic band gap (EBG) structure is shown in [18]. This antenna consists of dual circular polarization. However, the antenna has a superstrate dimension of 365 mm × 365 mm which is bulky for C band application. A wideband dual-polarized crossed-dipole antenna with parasitic crossed strip for base station applications has been presented where a pair of orthogonal crossed dipoles and two linear polarizations has been obtained and crossed strip is introduced to improve the impendence bandwidth and enhance the isolation [19]. A microstrip antenna array between two frequency selected surfaces (FSSs) was proposed for application in WLAN and LTE 4G systems [20]. Actually, FSS has been introduced to enhance the antenna performance. The parasitic elements are required to achieve notch within the pass-band of the antenna. While optimizing using genetic algorithm, two parasitic elements were included to increase the bandwidth performance of the antenna in [21, 22]. Finally, it can be concluded that slotted technique is the simplest structure to achieve miniaturized wide or multiband antenna. In this paper, a compact multislotted patch antenna has been designed for C/X band applications.

On the other hand, material science is becoming popular in RF (radio frequency) technology due to their outstanding deportment at high frequencies. Different material is used nowadays for constructing high frequency devices. Recently CM (composite material) and metamaterial are being used in high frequency technology and researchers are exploring new types of results. High dielectric constant of the substrate material is always desired for high frequency application to increased amounts of directivity and more centralized results. For a higher frequency resonator, the authors used a ferromagnetic compound as substrate material [23]. The material was chosen because the curie temperature of the material is greater than the room temperature, which indicates that the magnetic properties of the material remain intact at the operating temperature. The polarization characteristic of the resonating element is changed by changing the direction of the applied magnetic field. The oxidation behavior of zirconium diboride (ZrB_2_) based ultrahigh temperature ceramic composite was tested in high frequency plasma wind tunnel [24]. CMs were tested to show the dependence of composition on pressure, enthalpy, and heat flux. Carbon nanotube (CNT) composite material was used to design a nonmetallic resonator resonating at higher frequencies [25]. Antenna miniaturization using magnetodielectric and dielectric material was proposed in [26]. Glass-reinforced epoxy is used as RF and PCB (printed circuit board) material since 1968 by National Electrical Manufactures Association. Epoxy matrix reinforced by woven glass is the basic material for glass-reinforced epoxy. By changing the composition of the fiberglass and epoxy resin, the thickness of the substrate material can be changed and it is direction-dependent. By comparing the thermal stability of the glass-reinforced epoxy from different sources, it is shown that some are less stable and others are more thermally stable (higher lead-free processing temperatures) [27]. Bromine and phosphorus and aluminium hydroxide are chemical means for the glass-reinforced epoxy to be flame retardants. There is a lot of debate about when to use the glass-reinforced epoxy instead of high frequency laminates because of the more tangent loss. Now Rogers specially glass microfiber reinforced PTFE [13] and high permittivity ceramic polytetrafluoroethylene (PTFE) [14] composite are popularly used for their useful characteristics.

In this paper, glass microfiber reinforced PTFE characteristic is revised and a description of coupling within the material and at the surface of the material is also shown with related equation derivation and graphical view. A planar patch antenna is designed using glass microfiber reinforced PTFE material to demonstrate the behavior at radio frequency and compare with other reported materials by using simulation tool HFSS. New etching process other than chemical etching in glass microfiber reinforced PTFE is also introduced using LPKF drilling machine with 0.01 mm accuracy without any crack in the laminate. Finally, a simple, modified, rectangular-shaped, printed antenna that exhibits dual band characteristics has been designed. The reflection coefficients and far-field radiation pattern are presented. Return loss, radiation pattern, and gain measurements have also been conducted to verify and validate the performance of this new antenna design.

## 2. Antenna Design, Architecture, and Optimisation

**Figure 1:**
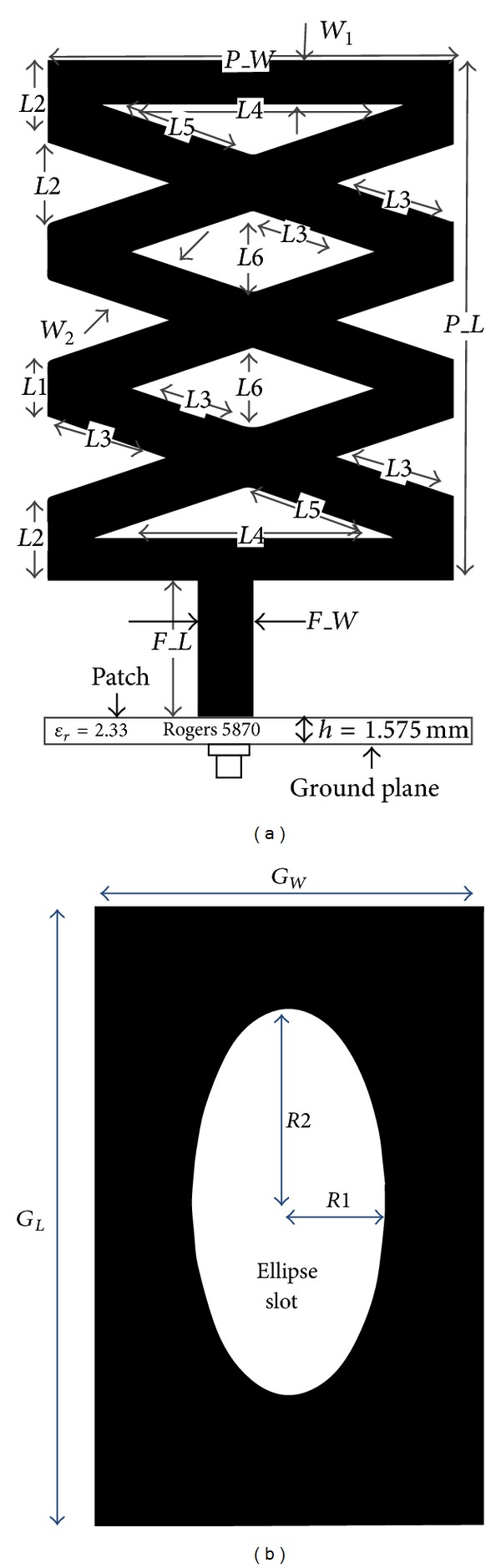
Proposed antenna geometry layout: (a) front and cross-sectional view and (b) bottom view.

The proposed, modified, rectangular-shaped, dual band antenna is illustrated in Figure 1. For the design studied here, the radiating element and feeding line are printed on the same side of the microstrip patch substrate, which has a thickness of 1.575 mm (*h*) and a dielectric constant of *ε*_*r*_ = 2.33 and tan*δ* = 0.002, while the other side is the ground plane of the antenna. The microstrip transmission line, for which the signal-strip thickness and length are denoted by *Wf* and *Lf*, respectively, is used to feed the antenna. The basic antenna structure begins as a rectangular-shaped patch. The radiating patch is created by cutting two diamond-shaped slots and two equatorial triangular slots in the middle as well as three triangular slots on both sides of the rectangular patch. An elliptical slot (for which the major radius and the ratio are *R*1 and *R*2, resp.) is etched on the ground plane of the proposed antenna. Different arm lengths of different slots (given by *L*1, *L*2, *L*3, *L*4, *L*5, and *L*6) and widths (*W*1, *W*2) have been optimized to obtain optimum outputs. The dual frequency, wide-impedance matching capability is enabled via the electromagnetic coupling effect of the ground plane to the feed line and the radiating patch. By properly selecting the antenna's geometric parameters numerically and experimentally, optimal wideband dual frequency antenna characteristics can be obtained while maintaining the antenna's compact size. Our experiment compares various dimensional parameters of the feed line to observe the resulting variations in the impedance bandwidths and the initial resonant frequencies of the proposed slot antennas. The optimal geometrical parameters of the proposed antenna are as follows: *P*_*L* = 38 mm, *P*_*W* = 30 mm, *L*1 = 4 mm, *L*2 = 6 mm, *L*3 = 9.48 mm, *L*4 = 22.71 mm, *L*5 = 12.11 mm, *L*6 = 6 mm, *W*1 = 3 mm, *W*2 = 4 mm, *Lf* = 10 mm, *Wf* = 4 mm, *G*_*L* = 48 mm, *G*_*W* = 30 mm, *R*1 = 17 mm, and *R*2 = 0.5.

## 3. Coupling Mechanism

**Figure 2:**
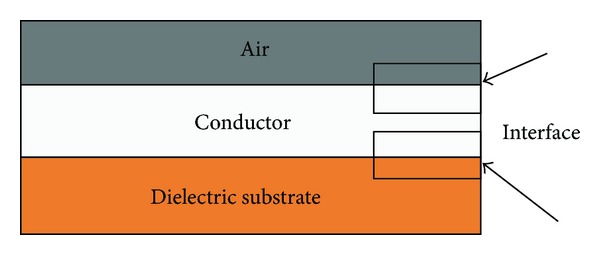
Resonator material Laris distribution.

The boundary conditions for the fields of the dielectric conductor and conductor air can be found using Maxwell's equations:

(1) $$\begin{array}{c} \oint_{s}D\cdot dS=Q_{\text{fce}}\\ \oint_{s}E\cdot dl=0, \end{array}$$

where *Q*_fce_ is the free surface charge. The conductor used in the resonator is copper “Cu” which is high in terms of electrical and thermal conductivity, resulting in having *ρ*_*c*_ → 0 and *σ* → *∞* which tends to a perfect conductor. The conductor and dielectric substrate interface of the resonator is shown in Figure 2. The electric field is *E* = 0 at the inside of the conductor. There is an electric field, normal to the conductor throughout the interface of the conductor-dielectric and conductor air (in the case of air, the dielectric constant is “1,” so the calculation for electric field is the same as for other dielectric substrates with different dielectric constants), and is external to the conductor.

The ground plane in Figure 1(b) has an elliptical slot in the middle, which indicates the resonator can be defined as an aperture type resonator. According to Babinet's principle, the complementary of that elliptical slot of the aperture slot of the aperture type resonant or radiation can be compared with the resonator radiation of a circular patch resonator. The principal is supported by the following equations [28]:

(2) $$\begin{array}{c} E_{\theta P}=H_{\theta c}\\ E_{\varphi P}=H_{\varphi c}\\ E_{\theta P}=-\frac{H_{\theta c}}{\eta_{0}^{2}}\\ H_{\varphi P}=-\frac{H_{\varphi c}}{\eta_{0}^{2}}, \end{array}$$

where *E*_*p*_ = electric field of circular patch, *η*_0_ = intrinsic impedance of free space, *E*_*c*_ = electric field of the complementary patch, *H*_*p*_ = magnetic field of circular patch, and *H*_*c*_ = magnetic field of complimentary patch. An elliptical slot is introduced in the middle of the rectangular ground plane shown in Figure 1(b) and in Figure 1(a) and three cross-slits are also embedded in the middle of the slotted patch. These cross-slits in the middle of the ground plane work as a finite element for the coupled lines.

## 4. Properties and Performance Analysis of Different Dielectric Substrate

Four different substrate materials, the properties of which are summarized below, are used in the simulation studies presented herein.

The FR4 substrate material consists of an epoxy matrix reinforced by woven glass. This composition of epoxy resin and fiber glass varies in thickness and is direction-dependent. One of the attractive properties of polymer resin composites is that they can be shaped and reshaped without losing their material properties. The composition ratio of the material is 60% fiber glass and 40% epoxy resin [29].

Taconic TLC laminates are engineered to provide a cost-effective substrate suitable for a wide range of microwave applications. TLC laminates offer superior electrical performance compared to thermoset laminates (e.g., FR-4, PPO, BT, polyimide, and cyanate ester). TLC's construction also provides exceptional mechanical stability. TLC laminates can be sheared, drilled, milled, and plated using standard methods for PTFE/woven fiberglass materials. The laminates are dimensionally stable and exhibit virtually no moisture absorption during fabrication. Taconic is a world leader in RF laminates and high speed digital materials, offering a wide range of high frequency laminates and prepregs. These advanced materials are used in the fabrication of antennas, multilayer RF and high speed digital boards, interconnections, and devices.

NeltecNX 9240 has superior mechanical and electrical performance, making it the material of choice for low-loss, high frequency applications, such as wireless communications. This material is specially designed for very low-loss antenna applications. The enhanced N9000 materials reduce passive intermodulation issues in antenna and high-power designs. The N9000 PTFE laminate system is the next generation material system designed for critical microwave components, antennas, power amplifiers, and subassemblies. Extensive R and D capability has produced passive intermodulation performance up to 25% better than other nonwoven or woven PTFE laminates currently available. Foil adhesion is 50–100% greater than competitive glass-reinforced PTFE laminates and 200–300% greater than ceramic loaded PTFE laminates on the market today. Superior mechanical and electrical performance makes the N9000 PTFE laminate system the material of choice for your lowest loss, high frequency applications.

RT/duroid 5870-filled PTFE composites are designed for exacting strip-line and microstrip circuit applications. The unique filler results in a low density, lightweight material that is beneficial for high-performance, weight-sensitive applications. The very low dielectric constants of such RT/duroid 5870 laminates are uniform from panel to panel and are constant over a wide frequency range. This substrate material is a high-frequency laminate and PTFE (polytetrafluoroethylene) composite amplified using glass microfibers. To increase the advantages of fiber reinforcement for circuit applications and circuit producers, these microfibers have been used aimlessly. The dielectric constant of this substrate material is lower than that of other products, and it is suitable for higher frequency bands, where dispersion and losses must be decreased because of the low dielectric loss. Due to the lack of extensive water absorption features, this substrate is typically used in heavy moisture environments. It is simply cut, machined, and sheared to shape, and it is impervious to all reagents and solvents, usually used in engraving printed circuit boards or plating holes and edges. Duroid 5870 has the lowest electrical loss of any amplified PTFE substrate material, lower absorption due to moisture is isotropic, and constant electrical characteristics are over frequency. These substrate materials have been used in circuitry for commercial airborne antenna systems, strip-line and microstrip circuits, lightweight feed networks, military radar systems, millimeter-wave applications, point-to-point digital radio antennas, and missile guidance systems. For these reasons, the RT/duroid 5870-filled PTFE composite substrate material was chosen for the proposed antenna design to achieve operation in the desired band.

Dielectric constant of substrates very much affects the antenna performance. The substrate, which has a low dielectric constant, will give better performance than the substrate which has a high dielectric constant. Loss tangent or dissipation factor also plays a part in antenna performance. The high dielectric material allows for a reduction of space but at the cost of higher moisture absorption level. Dielectric losses depend on the circuit configuration, dielectric constant, frequency, and loss tangent. Dielectric constant and loss tangent vary with operating temperature changes and levels of humidity. Dielectric constant values usually vary between 0 and 0.05% over a 100° Celsius for most PTFE based laminates. Loss tangent or dissipation factor can change significantly with moisture absorption as little as 0.25% of dielectric weight. Thus moisture absorption should be as low as possible. Dielectric materials cannot resist indefinite amount of voltage; with enough voltage applied, any insulating material will succumb to the electrical pressure and electron flow will occur. However, unlike the situation with conductors where the current is in a linear proportion to applied voltage (given a fixed resistance) current through an insulator is quite nonlinear; for voltage below a certain threshold level, virtually no electrons will flow, but if the voltage exceeds that threshold, there will be a rush of current. Once current is forced through an insulating material, the breakdown of that materials molecular structure has occurred. Hence volume resistivity and surface resistivity should be good. After breakdown, the material may or may not behave as an insulator any more, with the molecular structure having been altered by the breach. There is usually a “puncture” of the insulating medium where the electrons flowed during breakdown. The breakdown voltage should be higher. The thickness of an insulating material plays a role in determining its breakdown voltage and is otherwise known as dielectric strength.

**Figure 3:**
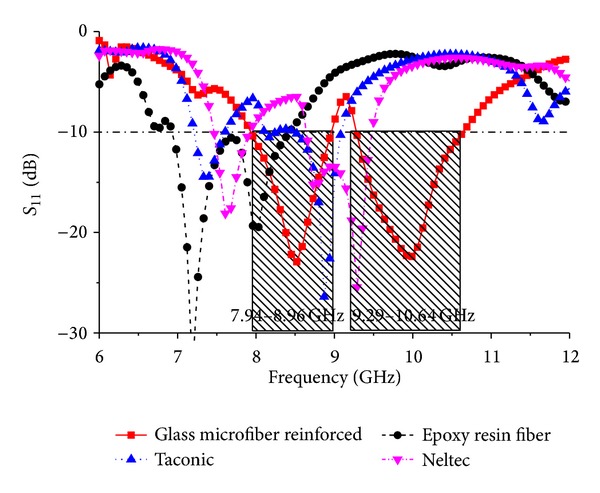
Reflection coefficient of the antenna for different substrate materials.

The effect of the different substrate material on the reflection coefficient of the proposed antenna is shown in Figure 3. It can be clearly observed that the proposed antenna offers a wider bandwidth and an adequate return loss value compared with other reported materials. Although the antenna with epoxy resin fiber material substrate provides a lower return loss value due to its higher dielectric constant, bandwidth is narrower, and the desired resonance is shifted. Moreover, the epoxy resin fiber loss tangent is higher compared to others. Other two material substrate materials have achieved resonance but low performance. The dielectric properties of the substrate material are tabulated in Table 1.

## 5. Parametric Study

**Figure 4:**
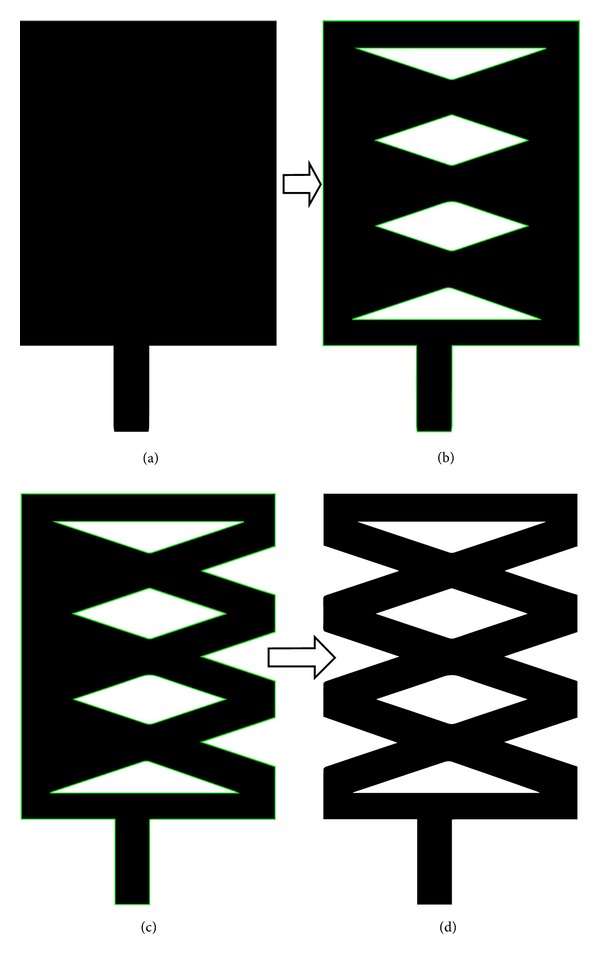
Antenna evaluation: (a) antenna 1, (b) antenna 2, (c) antenna 3, and (d) proposed antenna 4.

**Figure 5:**
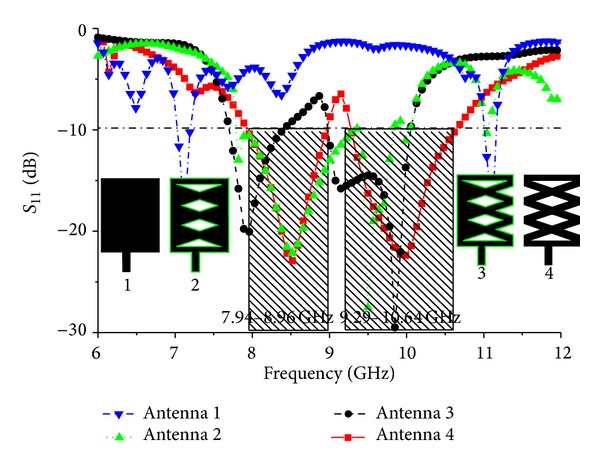
Simulated reflection coefficient of different values for different types of antenna.

A parametric study was performed to observe the effects of the proposed antenna parameters. The effects of the different parameters on the reflection coefficient were also observed. Firstly, design evaluation of the proposed patch antenna and the corresponding return losses are shown in Figures 4 and 5, respectively. The antenna's geometrical parameters have been evaluated with the aid of simulation software HFSS.

The reflection coefficients and the frequency dependence of these different shapes are shown in Figure 5. As shown in Figure 4(a), antenna 1 has a rectangular shape that exhibits three resonances and a smaller bandwidth, while antennas 2 and 3 display the same type of degenerate modes. The proposed shape shown in Figure 4(d) gives the desired impedance bandwidth. Because the proposed antenna operates in two different bands, the modified microstrip line is adjusted carefully to achieve good impedance matching in both operating bands.

**Figure 6:**
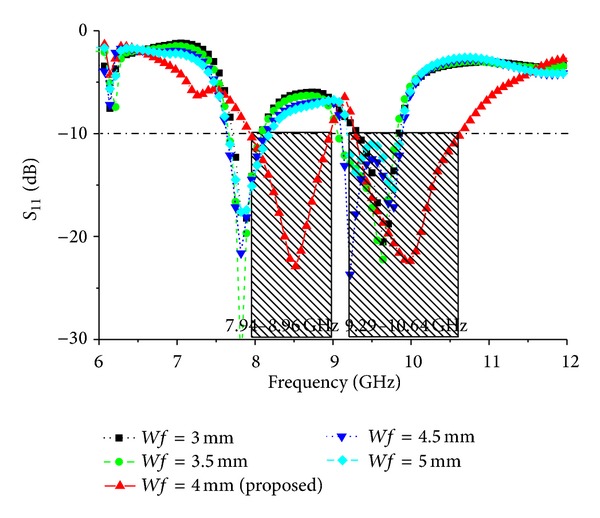
Simulated return loss of different values of feed width *Wf*.

**Figure 7:**
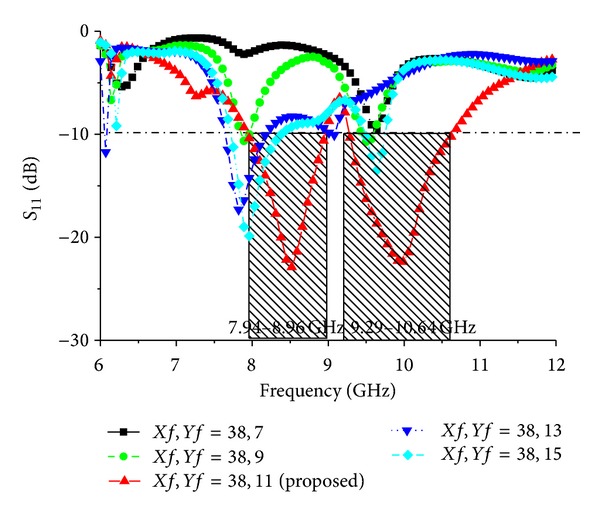
Simulated return loss for the microstrip feed line positions *Xf* and *Yf*.

**Figure 8:**
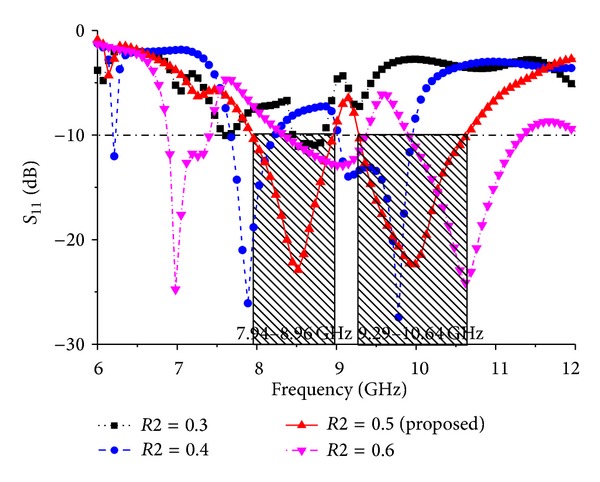
Simulated return loss for the different values of ground plane elliptical slot ratio *R*2.

**Figure 9:**
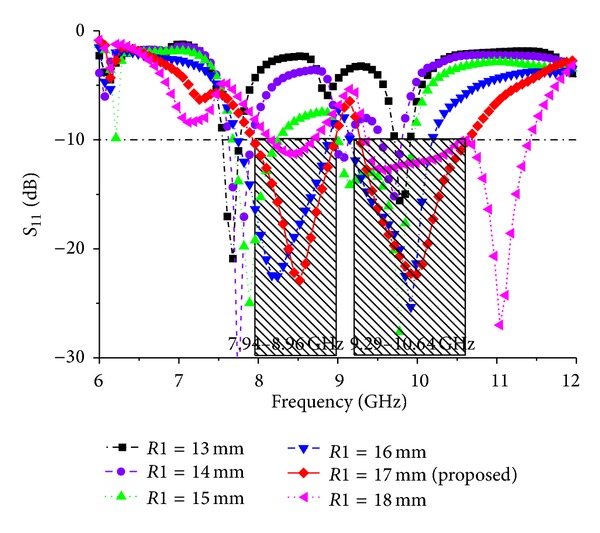
Simulated return loss for the different values of ground plane elliptical slot radius *R*1.

Different parameter values are investigated for *Wf*, feed position (*Xf* and *Yf*), and elliptical slot major radius *R*1 and ratio *R*2.  Figure 6 shows the simulated return loss of the antenna for different values of *Wf* and all other parameters as fixed as follows: *P*_*L* = 38 mm, *P*_*W* = 30 mm, *L*1 = 4 mm, *L*2 = 6 mm, *L*3 = 9.48 mm, *L*4 = 22.71 mm, *L*5 = 12.11 mm, *L*6 = 6 mm, *W*1 = 3 mm, *W*2 = 4 mm, *Lf* = 10 mm, *G*_*L* = 48 mm, *G*_*W* = 30 mm, *R*1 = 17 mm, and *R*2 = 0.5. When the values of *Wf* are reduced, the bandwidth decreases; however, when the values of *Wf* are increased from 4 mm, the bandwidth also decreases. According to Figure 6, the optimum strip line is *Wf* = 4 mm. The effect of the feed position, indicated by *Xf* and *Yf*, was also evaluated.  Figure 7 presents the simulated return loss for different values of *Xf* and *Yf*, showing that the chosen feed position affects the resonance and bandwidth of the antenna and that this position is crucial for obtaining efficient coupling. Finally, the influence of the elliptical slot radius and ratio was investigated when all other values are optimized as follows: *P*_*L* = 38 mm, *P*_*W* = 30 mm, *L*1 = 4 mm, *L*2 = 6 mm, *L*3 = 9.48 mm, *L*4 = 22.71 mm, *L*5 = 12.11 mm, *L*6 = 6 mm, *W*1 = 3 mm, *W*2 = 4 mm, *Lf* = 10 mm, *G*_*L* = 48 mm, *G*_*W* = 30 mm, *R*1 = 17 mm, and *R*2 = 0.5. Several simulations were carried out to determine the optimal radius that gives the largest bandwidth. These results are shown in Figures 8 and 9. For these curves, the matching bandwidth decreases and the resonant frequency shifts when the radius decreases or increases from the optimum value of *R*1 = 17 mm and ratio *R*2 = 0.5.

**Figure 10:**
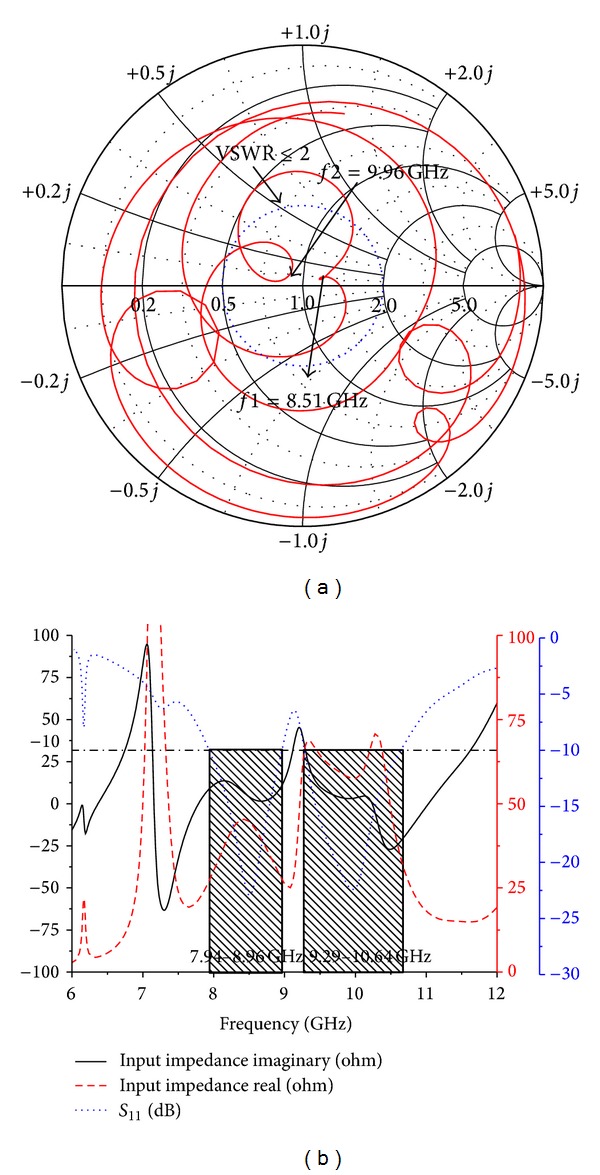
Simulated (a) Smith chart and (b) input impedance for the proposed dual band antenna.

The tuning parameters of the matching network have been studied carefully to achieve wideband operation. Adjusting the width of the 50 Ω microstrip line results in a trade-off between the impedance bandwidth and the initial frequency.  Figure 10 shows the Smith chart and input impedance of the proposed antenna. When the dimensions of the patch and the ground plane are changed, the coupling and the input impedance shift for the different resonant loops. The two tightest resonant loops are found at the center of the Smith chart of the proposed antenna. From Figure 10(a), the VSWR remains ≤2 at the center circle and, from the input impedance figure, the real and imaginary values are near to 50 Ω and zero.

**Figure 11:**
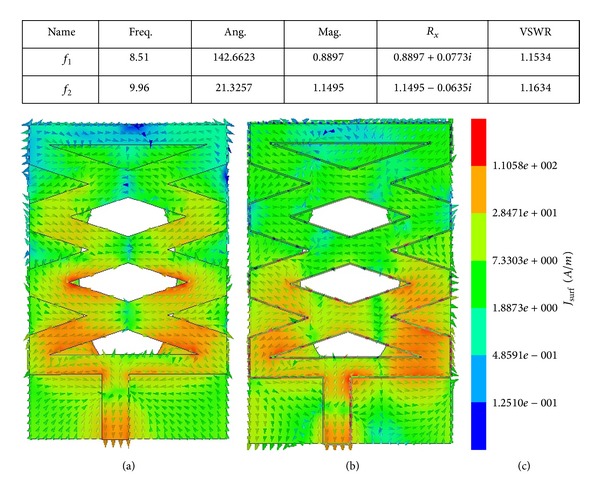
Surface current distributions at (a) 8.51 GHz and (b) 9.96 GHz (c) scale.

The near fields are examined numerically to better understand the antenna's mechanism.  Figure 11 depicts the top view of the electrical field at the two resonant frequencies. For these curves, at the lower resonant frequency, the near field is mainly concentrated in the microstrip patch line, the lower triangular slot, and the nearest diamond-shaped slot arm. At the upper resonant frequency, the electric field spreads from the feed patch and left and right lower triangular arm. It can be concluded that, at the upper frequency of the matching bandwidth, the radiation is due to the metallic patch.

**Figure 12:**
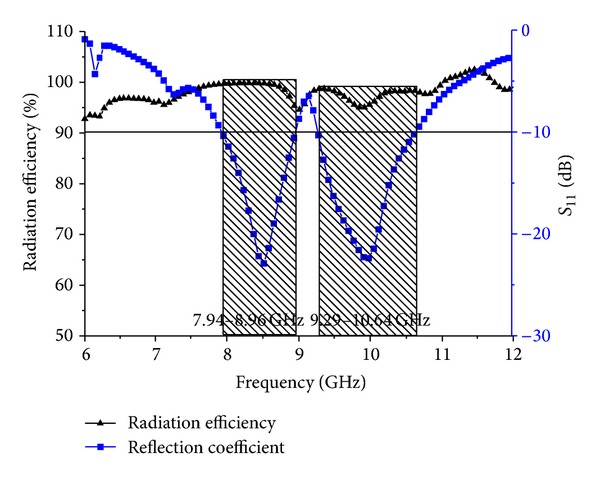
Proposed antenna radiation efficiency and *S*_11_.

**Figure 13:**
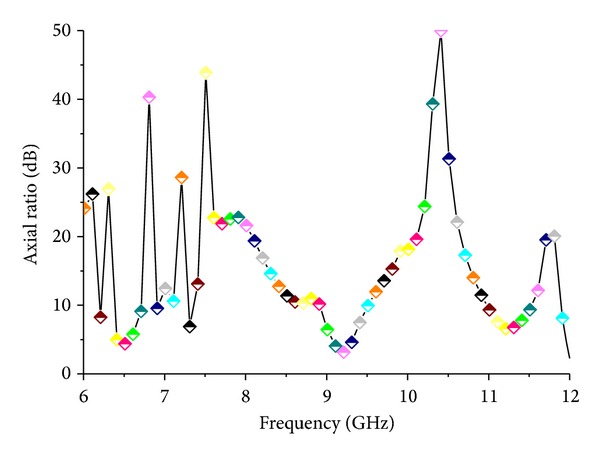
Axial ratio of the proposed antenna.

Figure 12 shows the radiation efficiency of the proposed antenna. The average radiation efficiency of the proposed antenna is approximately 98.18% at the lower band and 95.40% at the higher band.  Figure 13 depicts the axial ratio of the proposed antenna. Generally, the axial ratio is considered to determine antenna polarization. If the value of the antenna in the achieved band is less than 3 dB, then the antenna is called circular polarized. From the figure, it can be clearly stated that the value of the axial ratio is greater than 3 dB in the achieved band, which means the proposed antenna is linearly polarized.

## 6. Prototyping and Measurement Environment

**Figure 14:**
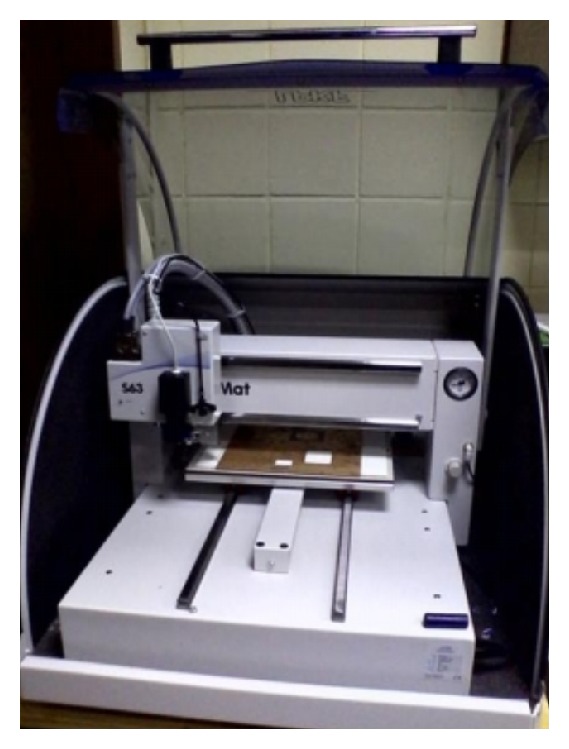
PCB prototyping machine (LPKF S63).

The technology for PCB prototyping is advancing exponentially with time. LPKF (S63), a prototyping machine company based in Germany, has developed a PCB prototyping machine that can prototype a dual sided PCB laminate of marginal length, width, and thickness. This machine can automatically reach out the unwanted copper layer from the RF laminate by using fast spin driller. The accuracy of the machine is as narrow as 0.01 mm. For both sides prototyping, a camera is attached to the driller so that calibration can be done before etching out both layers. The machine uses software named LPKF circuit pro to give command using a computer. For fast spin technology, if a hole is drilled on a glass microfiber reinforced PTFE laminate, the glass microfiber reinforced PTFE will not break down at the side of the hole or whatsoever. By using this machine, glass microfiber reinforced PTFE laminate material can easily be integrated with any type of RF module and any design specification using microstrip lines over glass microfiber reinforced PTFE laminate is possible.  Figure 14 shows the machine used for the fabrication of the antenna on glass microfiber reinforced PTFE material substrate.

**Figure 15:**
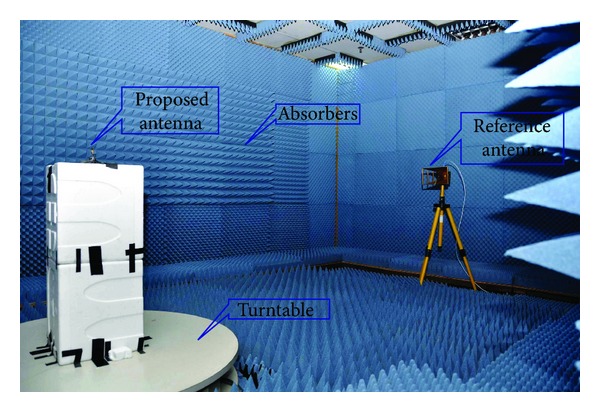
Illustrations of the anechoic chamber.

The prototype of the proposed antenna was measured in a standard far-field testing environment. An anechoic measurement chamber shaped like a rectangle 5.5 m × 4.5 m and 3.5 m high was used to measure the results for the parameters of the proposed antenna prototype. A double ridge guide horn antenna from AH systems incorporation was used as a reference antenna. A photograph of the UKM anechoic measurement chamber is shown in Figure 15. Pyramidal shaped electrically thick foam absorbers with less than −60 dB reflectivity at normal incidence were used on the walls, ceiling, and floor. A turntable of 1.2 m diameter was used to rotate the measuring antenna with specifications of a 1 RPM rotation speed and 360° rotation angle and connected with a 10 m cable between controllers. A vector network analyzer (VNA) with a range of 10 MHz up to 20 GHz was used for the measurements.

## 7. Experimental Results

**Figure 16:**
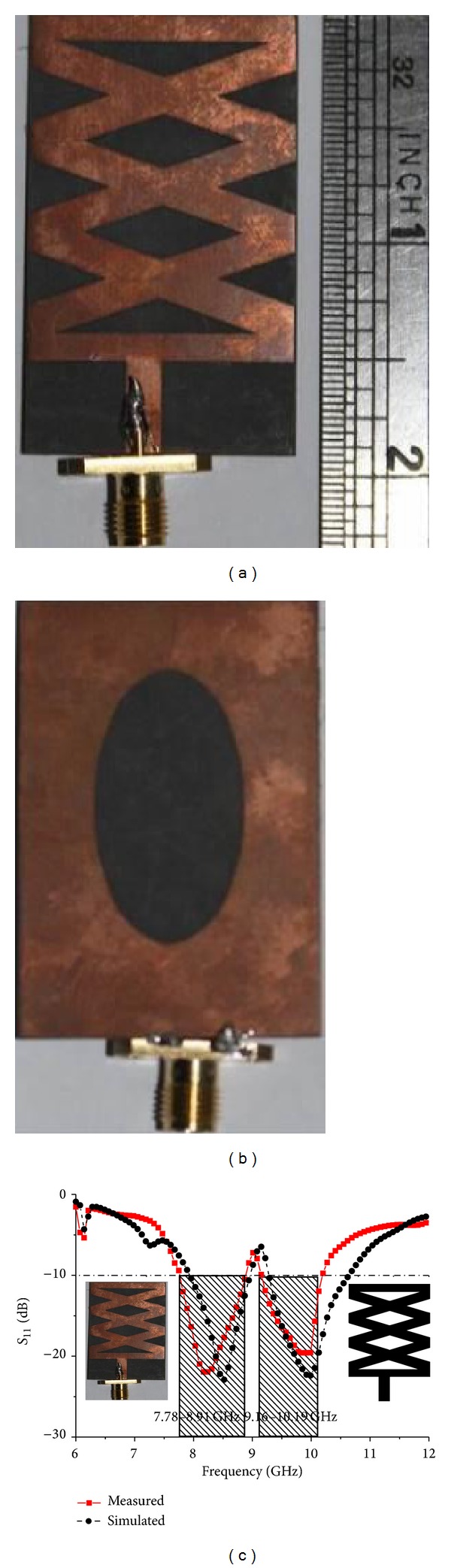
Photograph of the fabricated prototype: (a) front view; (b) back view; (c) simulated and measured reflection coefficients of the optimized antenna.

A prototype of the optimized antenna was fabricated and tested.  Figure 16 portrays the fabricated prototype of the antenna. The measured and simulated *S*_11_ parameters are shown in Figure 16(c). These graphs show good agreement between the simulated and measured results. The small shift between the simulated and measured results occurs because the SMA connector soldering was not included in the simulation. From the measured results, bandwidths of 11.58% (7.78–8.91 GHz) and 13.12% (9.16–10.19 GHz) where *S*_11_ < −10 dB at center frequencies of 8.25 GHz and 9.95 GHz, respectively, are achieved. The measured return loss bandwidth is slightly increased from its simulated value, but higher band impedance bandwidth is narrower than simulated value. The cutting slots in the patch and ground plane increased the current path, which increased the current intensity and as a result bandwidth increased.

**Figure 17:**
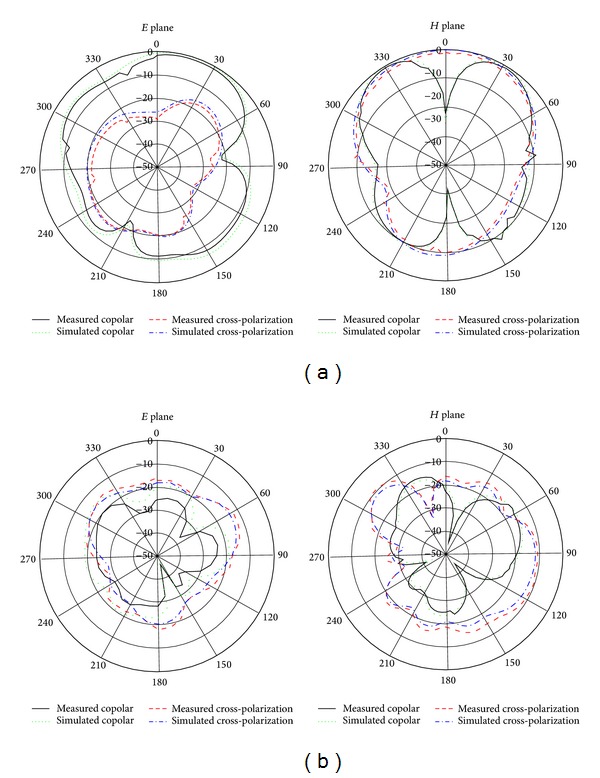
*E*- and *H*-plane radiation patterns of the proposed antenna at (a) 8.25 GHz and (b) 9.95 GHz.

The far-field radiation patterns for the proposed wideband dual frequency slotted antenna are also examined.  Figure 17 shows the measured co- and cross-polarised radiation patterns, including the horizontal (*E* plane) and vertical (*H* plane) polarization pattern for the antenna at a lower band of 8.25 GHz and an upper band of 9.95 GHz. These results demonstrate that the patterns are stable across the operating matching band. The slight asymmetry in the *H*-plane patterns is noteworthy and is due to the radiation of the microstrip line and the patch. Omnidirectional radiation patterns are observed in both the *E* and *H* planes. The obtained radiation patterns indicate that the proposed antenna delivers linear polarization, for which the level of cross-polarization is lower than that of copolarization in all of the simulated radiation patterns. When the radiation pattern of a microstrip antenna is symmetric and omnidirectional, it provides some reasonable benefits. One benefit is that the resonance does not shift for different directions, so a large amount of stable power is in the direction of the broadside beam. Another advantage is that the radiation pattern is more reliable on the operational bands. The level of cross-polarization at higher frequencies is comparatively higher than that at lower frequencies, which is desired due to the diffractions from the edges of the patch and ground plan. Additionally, the level of this cross-polarization is observed to be reduced by enhancing the slots on the ground plane; in addition, the triangle and diamond slot on the patch are also responsible for this effect. In this way, as will be discussed later, the enhancement of the slots is beneficial. The values of the other parameters are fixed. The results indicate that the radiation patterns are slightly shifted at the higher frequency because the distribution of the nonuniform phase is created on the proposed antenna. These radiation patterns are suitable for C and X band applications. The dimensions of the patch and the ground plane determine the radiation pattern degradation over the entire C and X bands. Thus, the sizes of the patch and the ground plane were selected carefully. If any parameter is changed, the resonant frequency is shifted. As a result, the radiation pattern is also changed from symmetric and omnidirectional to bidirectional or another type. Finally, the simulation results are close to measure results.

**Figure 18:**
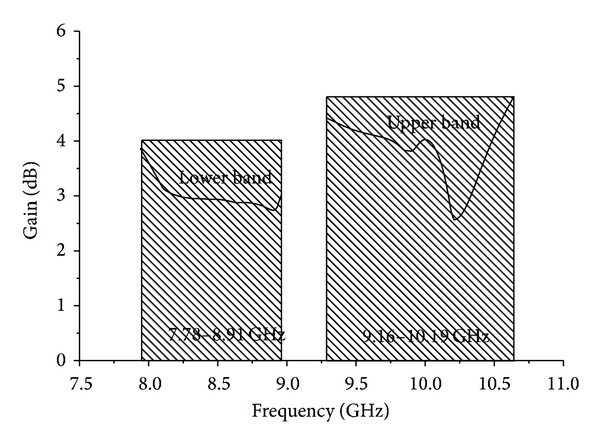
Achieved gains of the proposed antenna prototype.

Finally, Figure 18 shows the achieved gains of the proposed antenna at various frequencies across the two operating bands. A standard three-antenna system with two identical horn antennas was used for gain measurement. The gains of the two identical horn antennas are known, and a gain measurement system that follows well-known equations was used for three antennas. From the following equations, the gain of the three antennas (under test) can be calculated because the gains of two horn antennas are known, *R* is the distance between the two antennas, and *Pr* is the radiated power. Antenna 1 (horn) and antenna 2 (horn) are as follows:

(3) $$\begin{array}{c} G_{1}+G_{2}=2\log_{10}\left(\frac{4\pi R}{\lambda }\right)+10\log_{10}\left(\frac{P_{r2}}{P_{r1}}\right). \end{array}$$

Antenna 1 (horn) and antenna 3 (under test) are as follows:

(4) $$\begin{array}{c} G_{1}+G_{3}=20\log_{10}\left(\frac{4\pi R}{\lambda }\right)+10\log_{10}\left(\frac{P_{r3}}{P_{r1}}\right). \end{array}$$

Antenna 2 (horn) and antenna 3 (under test) are as follows:

(5) $$\begin{array}{c} G_{2}+G_{3}=20\log_{10}\left(\frac{4\pi R}{\lambda }\right)+10\log_{10}\left(\frac{P_{r3}}{P_{r2}}\right). \end{array}$$

For directivity *D*, the following equation [1] is used in which *U* is the radiation intensity and *P*_rad_ is the total radiated power:

(6) $$\begin{array}{c} D=\left(\frac{4\pi R}{\lambda }\right)\cdots . \end{array}$$

It could be noted that, in the lower band from 7.94 GHz to 8.96 GHz, the average gain was 2.96 dB and, in the upper band from 9.29 GHz to 10.64 GHz, the achieved average gain was 4.24 dB. Furthermore, the gain for the lower band was much less than for the upper band.

Proposed antenna characteristics are compared with some existing antenna in Table 2. It can be easily stated that the reported antennas are larger in size, lower in gain, or less efficient compared to proposed antenna. Also, the proposed slotted antenna performance is close to being more than six times better than conventional patch antenna.

## 8. Equivalent Circuit Model

**Figure 19:**
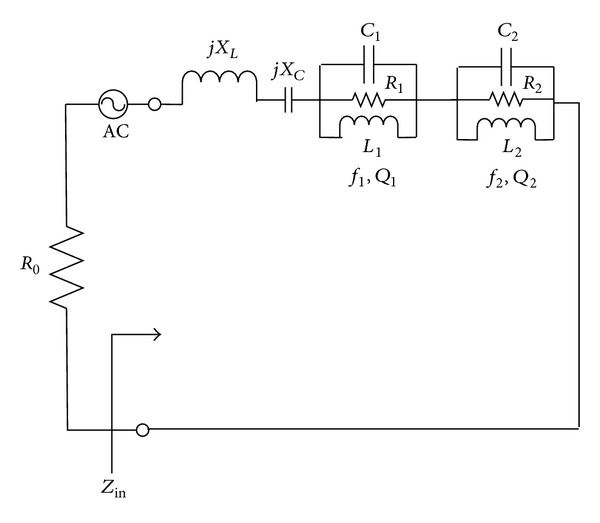
Equivalent circuit model of dual frequency proposed antenna.

Figure 19 indicates the proposed equivalent circuit consisting of two parallel resonance circuits for dual frequency wideband microstrip patch antenna as pictured in Figure 2. The two parallel resonance circuits are stated with two resonance frequencies *fc*1 and *fc*2 and unloaded *Q* factors *Q*1 and *Q*2, respectively. The input impedance of the equivalent circuit is defined as *Z*_in_(*f*), and the input reflection coefficient is specified with reference to the characteristic impedance *R*_0_.

The impedance of each resonant circuit is designated by

(7) $$\begin{array}{c} Z_{i}\left(f\right)=\frac{k_{i}R_{0}}{1+jQ\Omega_{i}\left(f\right)},\;i=1,2, \end{array}$$

where *k*_*i*_ is the coupling coefficient of the *i*th mode, and *Ω*_*i*_(*f*) = (*f*/*f*_*i*_) − (*f*_*i*_/*f*).

The series inductance and capacitance are presented by *X*_*L*_(*f*) = *X*_*Lo*_(*f*/*f*_*c*_) and *X*_*c*_(*f*) = −*X*_*co*_(*f*_*c*_/*f*), where *f*_*c*_ is the resonant frequency. Input impedance is as follows:

(8) $$\begin{array}{c} Z_{\text{in}}\left(f\right)=jX_{L}\left(f\right)+jX_{C}\left(f\right)+\overset{2}{\underset{i=1}{\sum }}Z_{i}\left(f\right). \end{array}$$

So, the real and imaginary parts of the impedance are

(9) $$\begin{array}{c} R_{c}\left(f\right)=\overset{2}{\underset{i}{\sum }}\frac{k_{i}R_{0}}{1+{\left(Q_{i}\Omega_{i}\left(f\right)\right)}^{2}},\\ X_{C}\left(f\right)=X_{L}\left(f\right)+X_{C}\left(f\right)-\overset{2}{\underset{i}{\sum }}\frac{k_{i}R_{0}Q_{i}\Omega_{i}\left(f\right)}{1+{\left(Q_{i}\Omega_{i}\left(f\right)\right)}^{2}}, \end{array}$$

respectively, with overall 8 unknown parameters to be decided: *Q*_1_, *Q*_2_, *f*_1_,  *f*_2_,  *k*_1_,  *k*_2_,  *X*_*L*_0__, and *X*_*C*_0__.

## 9. Conclusion

A novel miniaturized dual band antenna configuration containing triangular- and diamond-slotted patches with a slotted ground plane is projected. Glass microfiber reinforced PTFE and coupling mechanism for this antenna technology are also analyzed. Simulation is investigated by using different substrates for the same antenna design. The size of the patch in terms of the free-space wavelength at the lowest resonance frequency is 1.045 *λ*  ×  1.10 *λ*  ×  0.043 *λ*. In this design, a wide bandwidth has been obtained by adding different slots in the patch and ground plane. In addition, a parametric investigation was carried out to optimize the proposed design. For validation purposes, an antenna prototype has been fabricated and tested. Simulated and experimental results show that the proposed antenna can provide impedance bandwidths of 1130 MHz (7.78–8.91 GHz) and 1030 MHz (9.16–10.19 GHz) at center frequencies of 8.25 GHz and 9.95 GHz, respectively. Gains of 2.96 dB and 4.24 dB were achieved with radiation efficiencies of 98.18% at the lower band and 95.40% at the higher band. From the material analysis, it could be easily stated that glass microfiber reinforced material substrate performance is better than other reported materials. The proposed double-band antenna has a very simple, compact structure, which results in a simpler design and easier manufacturing and makes this antenna design very suitable for operations requiring the access points of C/X band application.

## Figures and Tables

**Table 1:** Properties of different substrates*.

Parameters	FR4_epoxy	Rogers RT/duroid 5870	Taconic	Neltec NY
Dielectric constant	4.6	2.33	2.4	2.4
Loss tangent	0.02	0.0012	0.0016	0.0016
Water absorption (%)	<0.25	0.02	<0.02	<0.02
Tensile strength	<310 MPa	450 MPa	—	—
Volume resistivity (Mohm·cm)	8 × 10^7^	2 × 10^7^	1 × 10^7^	1 × 10^7^
Surface resistivity (Mohm)	2 × 10^5^	3 × 10^7^	1 × 10^7^	1 × 10^7^
Breakdown voltage (kV)	55	>60	—	>60
Peel strength (N/mm)	9	5.5	12	12

*All information collected from the data sheet.

**Table 2:** Comparison between other existing antennas.

Article	Dimension (mm^3^)	Substrate and dielectric constant	Bandwidth (MHz)	Average gain (dB)
Conventional rectangular Figure 4(a)	38 × 30 × 1.575	Rogers 5870 *ε*_*r*_ = 2.33	150 (7040–7190) 130 (1102–1115)	1.11 5.43

[3]	12 × 14 × 1.6	FR4 *ε*_*r*_ = 4.4	112.02 (4039.99–4152.01) 127.7 (11066.15–11193.85) 558.36 (11720.82–12279.18) 681.6 (13829.2–14510.8)	Not mentioned

[4]	30.08 × 45.90 × 1	Rogers 5880 *ε*_*r*_ = 2.2	1100 (9600–1040)	6

[9]	55 × 60 × 2.4	FR4 *ε*_*r*_ = 4.4	300 (4100–4400) 1090 (8450–1054)	Not mentioned

[12]	DR is 12.8 × 7.3 × 6.35 Not mentioned	12.94 (*ε*_1_) and 3.38 (*ε*_2_)	280 (5960–6240), 490 (8055–8545)	5.9 5.55

[13]	30 × 40 × 1.575	Rogers 5870 *ε*_*r*_ = 2.33	45 (5670–5715) 110 (6490–6600) 150 (7610–7760) 230 (8810–9040)	2.20 2.91 5.96 4.30

[14]	12 × 16 × 1.905	Rogers 6010 *ε*_*r*_ = 10.2	1500 (5000–6500) 500 (9100–9600) 300 (1070–1100)	0.69, 3.52, 3.48

Proposed	38 × 30 × 1.575	Rogers 5870 *ε*_*r*_ = 2.33	1030 (9160–1019) 1028 (9034–1062)	2.96, 4.24
